# Epoxomicin, a Selective Proteasome Inhibitor, Activates AIM2 Inflammasome in Human Retinal Pigment Epithelium Cells

**DOI:** 10.3390/antiox11071288

**Published:** 2022-06-28

**Authors:** Iswariyaraja Sridevi Gurubaran, Maria Hytti, Kai Kaarniranta, Anu Kauppinen

**Affiliations:** 1 Department of Ophthalmology, Institute of Clinical Medicine, University of Eastern Finland, 70210 Kuopio, Finland; raja.sridevigurubaran@uef.fi (I.S.G.); kai.kaarniranta@uef.fi (K.K.); 2 Immuno-Ophthalmology, School of Pharmacy, Faculty of Health Sciences, University of Eastern Finland, 70210 Kuopio, Finland; maria.hytti@uef.fi; 3 Department of Ophthalmology, University of Eastern Finland, 70211 Kuopio, Finland; 4 Department of Ophthalmology, Kuopio University Hospital, 70029 Kuopio, Finland

**Keywords:** aging, age-related macular degeneration, oxidative stress, mitochondrial damage, reactive oxygen species, cytokines, IL-1β, antioxidants, inflammasomes, NLRP3, AIM2

## Abstract

Emerging evidence suggests that the intracellular clearance system plays a vital role in maintaining homeostasis and in regulating oxidative stress and inflammation in retinal pigment epithelium (RPE) cells. Dysfunctional proteasomes and autophagy in RPE cells have been associated with the pathogenesis of age-related macular degeneration. We have previously shown that the inhibition of proteasomes using MG-132 activates the NLR family pyrin domain containing 3 (NLRP3) inflammasome in human RPE cells. However, MG-132 is a non-selective proteasome inhibitor. In this study, we used the selective proteasome inhibitor epoxomicin to study the effect of non-functional intracellular clearance systems on inflammasome activation. Our data show that epoxomicin-induced proteasome inhibition promoted both nicotinamide adenine dinucleotide phosphate oxidase and mitochondria-mediated oxidative stress and release of mitochondrial DNA to the cytosol, which resulted in potassium efflux-dependent absence in melanoma 2 (AIM2) inflammasome activation and subsequent interleukin-1β secretion in ARPE-19 cells. The non-specific proteasome inhibitor MG-132 activated both NLRP3 and AIM2 inflammasomes and oxidative stress predominated as the activation mechanism, but modest potassium efflux was also detected. Collectively, our data suggest that a selective proteasome inhibitor is a potent inflammasome activator in human RPE cells and emphasize the role of the AIM2 inflammasome in addition to the more commonly known NLRP3 inflammasome.

## 1. Introduction

**Figure 1:**
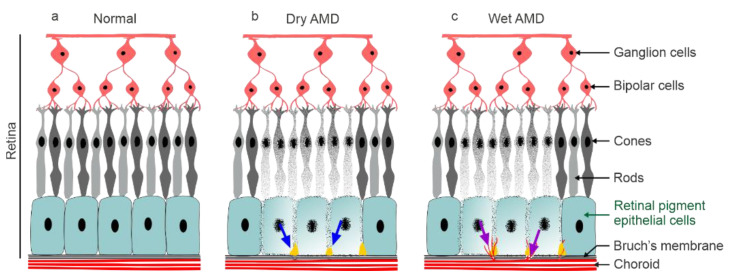
(**a**) A schematic representation of the normal retina; (**b**) dry AMD represents the accumulation of drusen deposits (blue arrow) and the degeneration of RPE cells; (**c**) changes in dry AMD, with choroidal neovascularization (purple arrow) appearing in wet AMD.

Age-related macular degeneration (AMD) is the leading cause of vision loss with increasing prevalence among people older than 60 years [1]. By destroying the patients’ central vision, it reduces their quality of life and causes a significant socioeconomic burden [2]. Genetic and environmental risk factors contributing to increased oxidative stress, mitochondrial damage, accumulation of oxidized proteins and lipids, and low-grade chronic inflammation within the retinal pigment epithelium (RPE) predispose the adult population to age-related macular degeneration (AMD) at advanced age [3,4,5,6,7]. The dysfunctionality of RPE cells is followed by the loss of photoreceptor cells (PRCs) in the macula, a specialized region in the retina responsible for the fine-tuning of central vision [8,9]. Although understanding of the molecular mechanisms of AMD has increased, there is no cure for the disease, and only wet AMD can currently be decelerated by intravitreal injections of anti-vascular endothelial growth factor (VEGF) drugs [10]. Dry AMD with retinal atrophy is more common (85–90%) in comparison to wet AMD, which is characterized by neovessel formation from the choroid into the retina (Figure 1) [11].

Besides contributing to the blood–retina barrier, RPE cells play crucial roles in the retina by secreting growth factors, controlling the transport of nutrients into and waste products out of the retina [12]. RPE cells are also involved in the immune defense of the retina by interacting with microglia cells, activating the complement system, and releasing molecules such as cytokines, chemokines, and neurotrophic factors [13,14,15]. Moreover, RPE cells phagocytize 10% of the PRCs outer segment (POS) in the process called heterophagy [16]. Heterophagy utilizes the endolysosomal autophagy system that also degrades exhausted organelles and protein aggregates [16]. Prior to aggregation, damaged, misfolded, and obsolete proteins can become degraded by proteasomes that serve as a major intracellular degradation system [17]. Both proteasomes and autophagy maintain cellular homeostasis, and dysfunctional intracellular clearance aggravates inflammation in RPE cells and contributes to the progression of AMD [4,18,19,20,21,22,23].

We have previously demonstrated NLRP3 inflammasome activation in human RPE cells upon inhibition of proteasomes and autophagy using the reversible, cell-permeable proteasome inhibitor MG-132 and the vacuolar H+ ATPase (V-ATPase) inhibitor bafilomycin A1 (BafA), [24], respectively [25,26]. However, several studies attribute MG-132 as a multifunctional compound capable of inhibiting certain lysosomal cysteine proteases and calpains, i.e., calcium-dependent, non-lysosomal cysteine proteases [27,28,29]. Therefore, a more specific protease inhibitor would be beneficial in studying the role of dysfunctional proteasomes in RPE cells. In this study, we used the potent and selective proteasome inhibitor epoxomicin [28,30] to inhibit proteasomes in human ARPE-19 cells and evaluated its effects on inflammasome activation.

## 2. Materials and Methods

### 2.1. Cell Culturing and Exposures

ARPE-19 cells (American Type Culture Collection (ATCC), Manassas, VA, USA) were grown in humidified conditions with 5% CO_2_ at 37 °C. The cells were maintained in DMEM/F-12, a 1:1 mixture of Dulbecco’s modified essential medium (DMEM) and Ham’s F-12 medium, (Life Technologies, Carlsbad, CA, USA) with 10% inactivated fetal bovine serum (FBS; Thermo Fisher Scientific, Waltham, MA, USA), 2 mM L-glutamine, and 100 units/mL of penicillin and streptomycin (all from Lonza, Basel, Switzerland).

For experiments, ARPE-19 cells were seeded on 12-well plates at the density of 2 × 10^5^/mL for 3 days to achieve 85–90% confluency. Before treatments, cell cultures were washed twice using serum-free DMEM/F-12 medium and primed with IL-1α (4 ng/ml, R&D Systems, Abington, UK) in serum-free medium for 24 h. Thereafter, MG-132 (5 µM/mL, Calbiochem, San Diego, CA, USA) or epoxomicin (0.5 µM/mL, BostonBiochem, MA, USA) was added for 24 h or 48 h, after which cells were further treated with Bafilomycin A1 (BafA; 50 nm/mL, Sigma-Aldrich, Munich, Germany) for 24 h.

To determine the effects of antioxidants on cell responses to MG-132, epoxomicin, and BafA, N-acetyl cysteine (NAC, 10 mM) or (2R,4R)-4-Aminopyrrolidine-2,4-dicarboxylic acid (APDC, 50 µM), both from Merck KGaA (Darmstadt, Germany), was added 5 min, and MitoTempo (100 µM, Merck KGaA, Darmstadt, Germany) was added 1 h before MG-132 or epoxomicin.

For studying the role of potassium (K^+^) efflux on inflammasome activation, 50 mM potassium chloride (KCl, Scharlab, S.L., Barcelona, Spain) was added to cell cultures, along with IL-1α, for 24 h prior to the addition of MG-132 or epoxomicin for 48 h and BafA for 24 h.

The role of caspase-1 was studied by adding a 50 µM caspase-1 inhibitor (Calbiochem, San Diego, CA, USA) to cell cultures 1 h prior to the treatment with MG-132 or epoxomicin. 

### 2.2. Sample Preparation

Medium samples were collected to microtubes after the BafA treatment and centrifuged at 3.8 × 10^2^× *g* for 10 min. Supernatants were transferred into clean tubes and stored at −70 °C until analyzed. 

### 2.3. Cell Viability Assay

In order to determine cellular viability upon exposure, lactate dehydrogenase (LDH) levels were measured from medium samples using a commercial kit (Promega, Madison, WI, USA) according to the manufacturer’s protocol.

### 2.4. ELISA Assay

Human interleukin-1β (IL-1β) (BD Bioscience, San Diego, CA, USA) was measured from medium samples using the enzyme-linked immunosorbent assay (ELISA) technique, as described previously [26]. Absorbance values were measured at the wavelength of 450 nm with the reference wavelength of 655 nm using a spectrophotometer (Bio-Rad, Model 550, Hercules, CA, USA).

### 2.5. ROS Measurements

To measure the levels of reactive oxygen species (ROS), 10 µM of ROS-sensitive fluorescent dye 2′,7′-dichlorodihydrofluorescein diacetate (DCFDA; Thermo Fisher Scientific) was added 5 min prior to epoxomicin or MG-132 treatment. After incubation with DCFDA for 1 h, the cell cultures were washed twice with Dulbecco’s phosphate-buffered saline (DPBS, Gibco^®^, Thermo Fisher Scientific), and the fluorescence signal was detected at the excitation and emission wavelengths of 488 nm and 528 nm, respectively, using a fluorometer (Cytation 3, BioTek Instruments, Inc., Winooski, VT, USA).

### 2.6. siRNA Treatments

ARPE-19 cells were seeded at the concentration of 1.5 × 10^5^ cells per well onto 12-well plates. The sub-confluent (60–80%) cultures were washed, and fresh medium was added without antibiotics. Cells were exposed to Silencer^®^ Select NLRP3 siRNA (ID: s41556) or Silencer^®^ Select AIM2 siRNAs (IDs: s18092) and Silencer^®^ Select negative control (Cat. no. 4390843, all from Ambion by Life Technologies) at 10 pmol per ml. Mixtures of siRNAs and transporter were prepared in an Opti-MEM medium (Gibco, Grand Island, NY, USA) and transfected using Lipofectamine® RNAiMAX Reagent (Invitrogen, Van Allen Way Carlsbad, CA, USA) according to the manufacturer’s instructions. The transfected cells were incubated for 24 h and exposed to epoxomicin or MG-132 and BafA in a fresh medium, as described above. 

### 2.7. mtDNA Extraction and Measurement

mtDNA extraction and measurement were made using the adopted and modified protocol from Bronner et al. [31]. The cells were seeded at the concentration of 2 × 10^6^ cells per well onto 10 cm culture plates, followed by priming and exposure to epoxomicin or MG-132 in serum-free media. Cells were washed once with DPBS (Gibco^®^, Thermo Fisher Scientific) and lysed using 1% Nonidet^®^ P 40 Substitute (NP-40) (Cat.no. M158, VWR Life Science). The mtDNA was isolated in microtubes using a commercial kit (Macherey-Nagel, Düren, Germany). The amplification and measurement of mitochondrial cytochrome b (mt-CYB) and house-keeping gene GAPDH (Table 1) were made using Quantstudio^TM^ 5 System and SYBR^®^ Green chemistry (both from Applied Biosystems). The thermo-cycling program consisted of 40 cycles at 95 °C for 30 s and at 60 °C for 60 s, with an initial cycle at 95 °C for 10 min. The melting curve analysis was performed at 72 °C for 30 s and at 95 °C for 60 s, as well as at 60 °C for 30 s and at 95 °C for 30 s. Finally, mtDNA was quantified and analyzed using the 2^ΔΔCt^ method.

### 2.8. Statistical Analysis

All statistical analyses were conducted using GraphPad Prism 8 (GraphPad Software, San Diego, CA, USA). Pairwise comparisons between groups were performed using the Mann–Whitney U test, and *p*-values of 0.05 or less were considered significant. 

## 3. Results

### 3.1. Epoxomicin Is a More Potent Inducer of IL-1 Release from ARPE-19 Cells than MG-132

**Figure 2:**
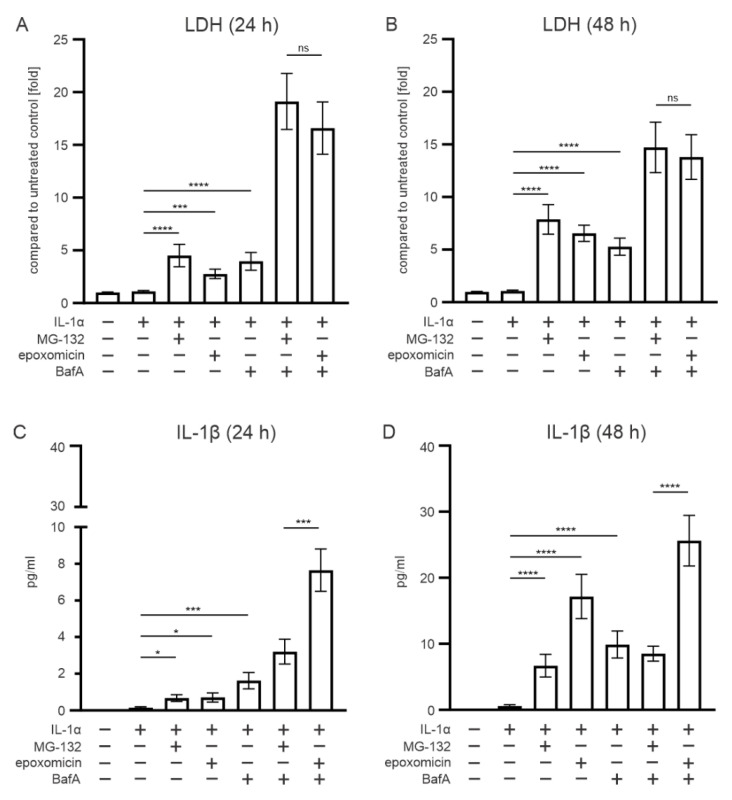
The effect of proteasome inhibitors epoxomicin or MG-132 with and without BafA on (**A**,**B**) cell viability and (**C**,**D**) IL-1β production in ARPE-19 cells. Data of LDH and IL-1β were combined from nine independent experiments, with four parallel samples in each group. The results are presented as mean ± SEM. * *p* ≤ 0.05, *** *p* ≤ 0.001, **** *p* ≤ 0.0001; ns, not significant.

In order to simulate our previous model with dysfunctional cellular clearance in RPE cells [26] more precisely, we also added BafA to the epoxomicin exposure. LDH measurement revealed that epoxomicin treatment significantly compromised the cell viability, compared with IL-1α-primed cells at 24 h and 48 h time points (Figure 2A,B). The reduction in cell viability was at the same level in cells exposed to MG-132 (Figure 2A,B). In addition, the cytokine IL-1β levels were significantly increased in epoxomicin- or MG-132-treated cells, compared with IL-1α-primed cells at both time points (Figure 2C,D). However, at the 48 h time point, IL-1β levels were approximately twofold higher in epoxomicin-treated cells, compared with MG-132 treated cells (Figure 2D). The addition of BafA further reduced the cell viability in epoxomicin-treated cells (Figure 2A,D), and similar effects were observed in MG-132-treated cells. At the 24 h and 48 h time points, the presence of epoxomicin with BafA treatment increased the IL-1β levels by ca. two- and threefold, respectively, in comparison to MG-132 with BafA treatment (Figure 2C,D). Collectively, our data suggest that epoxomicin is a more potent IL-1β inducer than MG-132 while showing a similar effect on RPE cell viability.

### 3.2. The Effect of Epoxomicin on Cell Viability and IL-1β Release Are Mediated by Caspase-1

**Figure 3:**
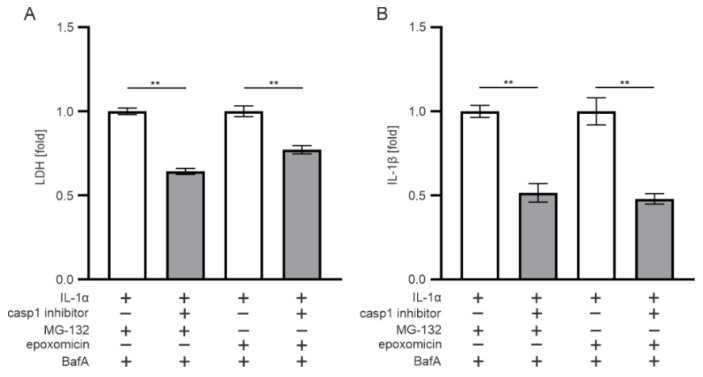
The effect of caspase-1 (casp1) inhibition on (**A**) cell viability and (**B**) IL-1β secretion in ARPE-19 cells exposed to epoxomicin or MG-132 and BafA. Data of LDH and IL-1β were combined from three independent experiments with four parallel samples in each group. The results are presented as mean ± SEM. ** *p* ≤ 0.01.

Since IL-1β is matured by the inflammasome-related caspase-1 enzyme, we used caspase-1 inhibitors to study whether they contribute to epoxomicin-induced responses. The inhibition of caspase-1 significantly increased the viability of RPE cells upon exposure to epoxomicin or MG-132 and BafA, compared with cells lacking the caspase-1 inhibitor (Figure 3A). In addition, the concurrent IL-1β levels were significantly reduced (Figure 3B). These data suggest that the rupturing of the epoxomicin-induced cell membrane and IL-1β release from ARPE-19 cells were caspase-1-mediated processes.

### 3.3. AIM2, but Not NLRP3, Participates in the Epoxomicin-Induced Inflammasome Signaling

**Figure 4:**
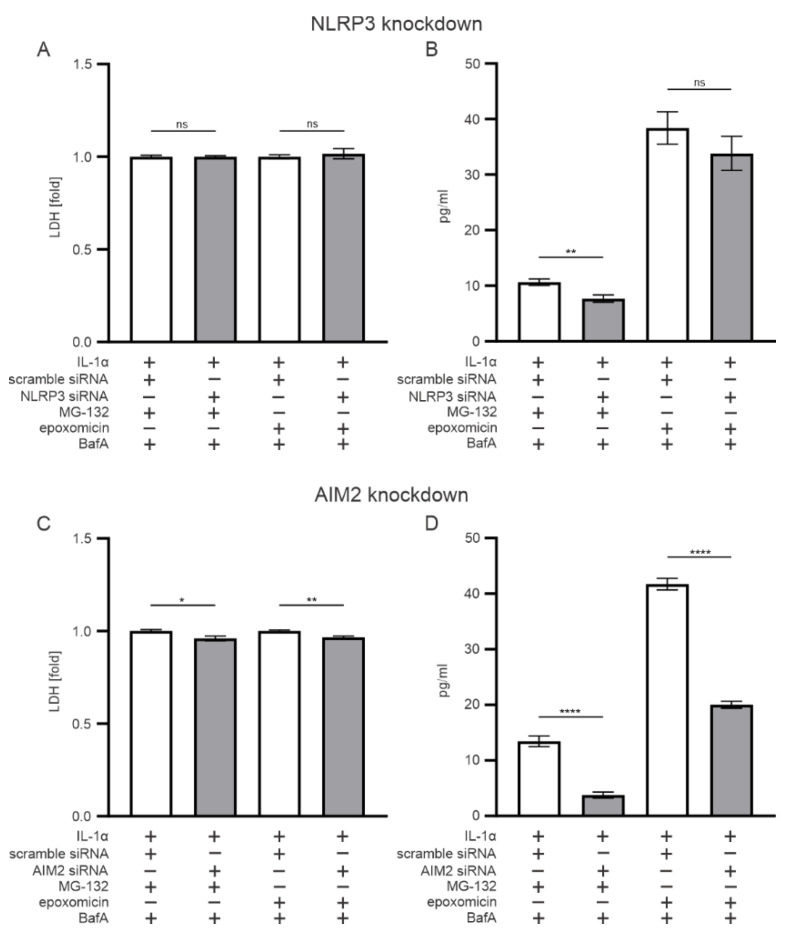
The role of (**A**,**B**) inflammasome receptors NLRP3 and (**C**,**D**) AIM2 on (**A**,**C**) cell viability and (**B**,**D**) IL-1β levels in ARPE-19 cells. Data of LDH and IL-1β were combined from three independent experiments with four parallel samples in each group. The results are presented as mean ± SEM. * *p* ≤ 0.05, ** *p* ≤ 0.01, **** *p* ≤ 0.0001; ns, not significant.

In our previous study, we showed NLRP3 inflammasome activation by MG-132 in human RPE cells [26]. In the present study, we tested whether the same applies also to epoxomicin. We used NLRP3-specific siRNA that did not compromise cell viability in epoxomicin- or MG-132-and-BafA-treated cells when compared with scrambled siRNA (Figure 4A). The NLRP3 siRNA significantly reduced the IL-1β release from IL-1α-primed ARPE-19 cells exposed to MG-132 and BafA but not from cells in which epoxomicin replaced MG-132 (Figure 4B). The knockdown of AIM2 significantly increased cell viability (Figure 4C) and significantly reduced IL-1β levels in cells treated either with epoxomicin or MG-132 and BafA (Figure 4D). Together, these results suggest that epoxomicin treatment unambiguously involves AIM2 inflammasome activation, whereas MG-132 treatment activates both NLRP3 and AIM2 inflammasomes.

### 3.4. Cytosolic mtDNA Is Especially Associated with Epoxomicin-Induced IL-1β Production in RPE Cells

**Figure 5:**
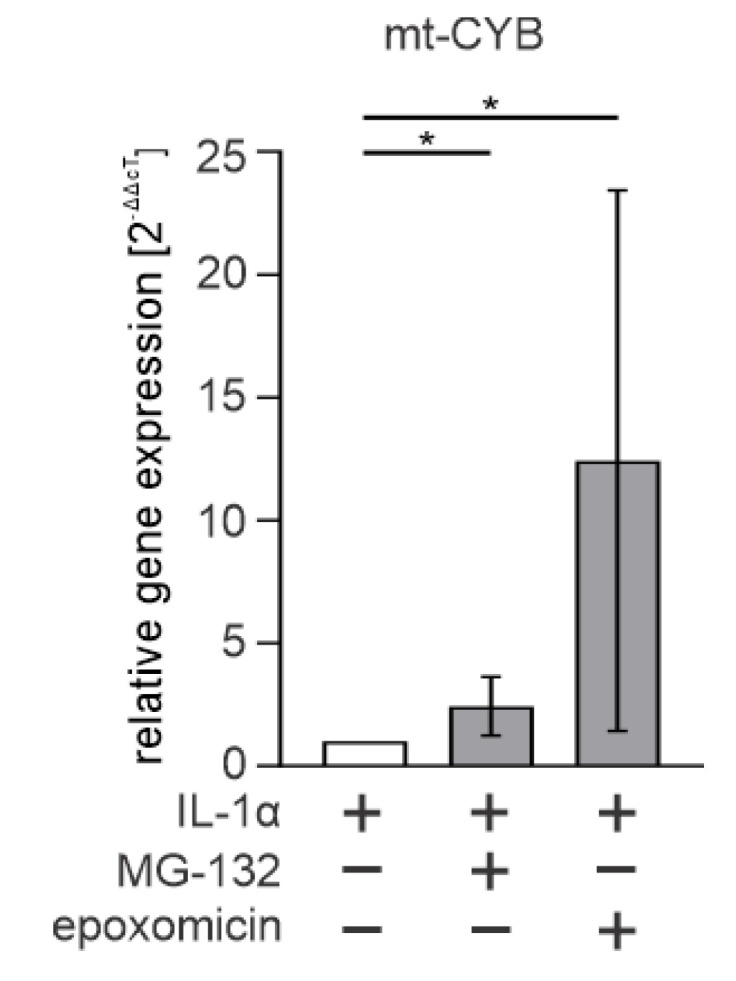
The expression of cytosolic mt-CYB in MG-132 or epoxomicin-treated ARPE-19 cells. Data were combined from four independent experiments, with at least two parallel samples in each group. The results are presented as mean ± SEM. The results are presented as mean ± SEM. * *p* ≤ 0.05.

Since the AIM2 inflammasome is a known dsDNA sensor [32], we tested whether cytosolic mtDNA plays a role in epoxomicin-induced inflammasome signaling. IL-1α-primed ARPE-19 cells exposed to epoxomicin had ca. 12 times more mtDNA in their cytosol when compared with IL-1α-primed cells (Figure 5). The increase in mtDNA levels by epoxomicin was ca. fivefold higher than that by MG-132 treatment (Figure 5). Altogether, our data show that increased mtDNA levels in the cytosol are associated with the AIM2 inflammasome activation in ARPE-19 cells upon exposure to epoxomicin. 

### 3.5. K^+^ Efflux Contributes to the IL-1β Release in Epoxomicin-Treated Human RPE Cells

**Figure 6:**
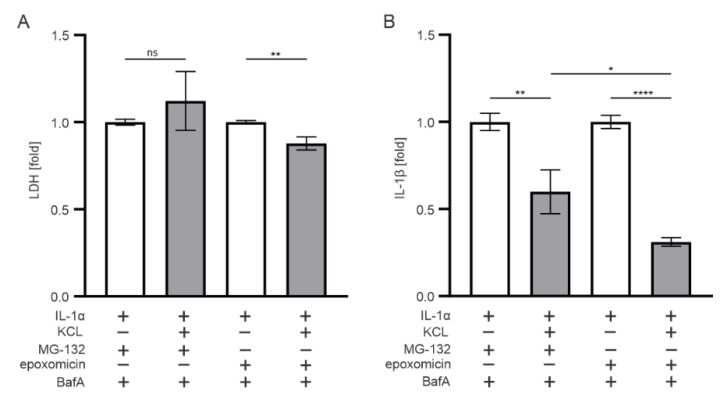
The effect of extracellular KCl on (**A**) cell viability and (**B**) IL-1β release in ARPE-19 cells upon exposure to epoxomicin or MG-132 and BafA. Data of LDH and IL-1β were combined from three independent experiments, with four parallel samples in each group. The results are presented as mean ± SEM. * *p* ≤ 0.05, ** *p* ≤ 0.01, **** *p* ≤ 0.0001; ns, not significant.

The role of K^+^ efflux in oxidative stress, mitochondrial damage, inflammasome activation, and cytokine production has been reported in various studies [33,34,35,36,37]. In our previous study, it was not responsible for the MG-132-and-BafA-induced inflammasome activation in ARPE-19 cells [38], but due to the observed differences in responses to MG-132 vs. epoxomicin, we tested the role of K^+^ efflux also upon exposure of ARPE-19 cells to epoxomicin. The inhibition of K^+^ efflux from ARPE-19 cells by KCl significantly increased cell viability in epoxomicin-and-BafA-treated cells, while the MG-132-and-BafA group did not show a similar effect (Figure 6A). Extracellular KCl also significantly reduced the IL-1β levels released from cells exposed to either epoxomicin or MG-132 together with BafA when compared with cells without KCl supplement (Figure 6B). IL-1β levels were ca. 2 times lower in epoxomicin-and-BafA-treated cells in comparison with those exposed to MG-132 instead of epoxomicin. The data suggest that K^+^ efflux is a more important player upon exposure of RPE cells to epoxomicin than to the unspecific proteasome inhibitor, MG-132.

### 3.6. Oxidative Stress also Contributes to the IL-1β Release Upon Exposure of RPE Cells to Epoxomicin

**Figure 7:**
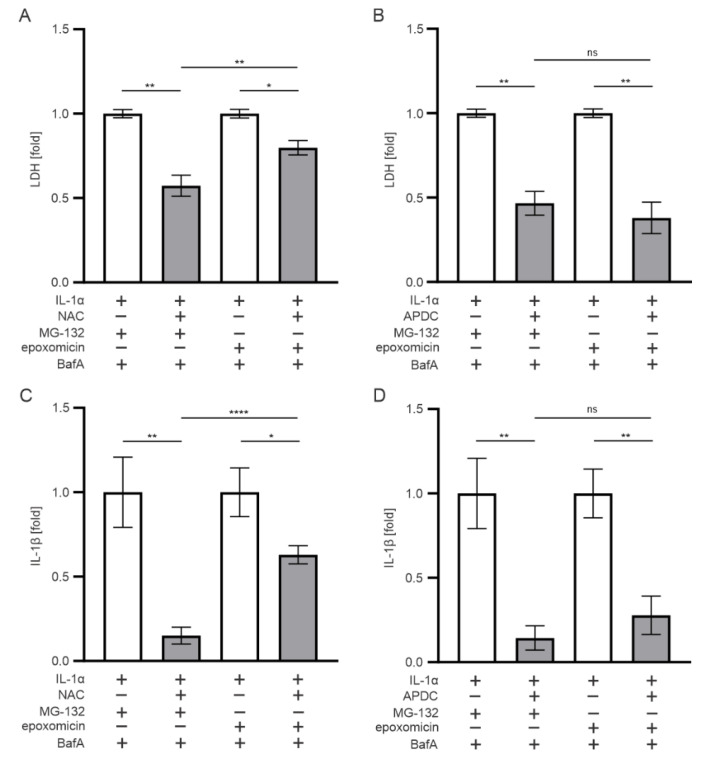
The effect of antioxidants on (**A**,**B**) cell viability and (**C**,**D**) IL-1β production in ARPE-19 cells. Data were combined from three independent experiments, with four parallel samples in each group. The results are presented as mean ± SEM. * *p* ≤ 0.05, ** *p* ≤ 0.01, **** *p* ≤ 0.0001; ns, not significant.

Dysfunctional intracellular clearance and the accumulation of waste material are known to cause oxidative stress, which was the main regulator of inflammasome activation in MG-132-and-BafA-treated RPE cells [38,39,40]. To examine the role of oxidative stress in the present study, we added NAC or APDC to cell cultures prior to the exposure of cells to epoxomicin or MG-132. The presence of the glutathione (GSH) precursor and potent antioxidant NAC [41] recovered cell viability (Figure 7A) and reduced the IL-1β secretion (Figure 7C) in epoxomicin-and-BafA-treated ARPE-19 cells when compared with otherwise similarly treated cells without NAC (Figure 7A,C). The effect of NAC was even stronger in cells exposed to MG-132 and BafA (Figure 7A,C). The addition of APDC, a potent inhibitor of NADPH oxidase [38,42], also significantly increased cell viability and reduced IL-1β release from epoxomicin or MG-132-and-BafA-treated cells (Figure 7B,D). In contrast to NAC, the effect of APDC was equal in cell cultures exposed either to epoxomicin or MG-132 (Figure 7B,D). Collectively, our data suggest that oxidative stress also participates in the epoxomicin-induced cytotoxicity and IL-1β maturation in human RPE cells, but its role is more prominent in cells exposed to MG-132. 

### 3.7. Mitochondria Contributes to the ROS Production upon Epoxomicin Exposure

**Figure 8:**
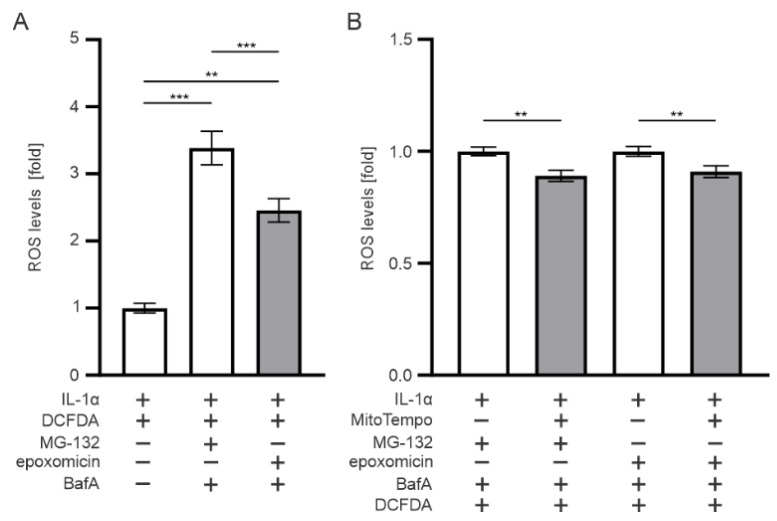
(**A**) ROS levels in APRE-19 cells exposed to epoxomicin or MG-132 with BafA; (**B**) cell viability and ROS levels under similar conditions with MitoTempo. Data of ROS and LDH were combined from at least four independent experiments, with four parallel samples in each group. The results are presented as mean ± SEM. ** *p* ≤ 0.01, *** *p* ≤ 0.001.

Since antioxidants NAC and APDC increased ARPE-19 cell viability and reduced IL-1β levels, we measured the ROS levels and tested mitochondria for the potential source of ROS in epoxomicin-treated cells. Exposure of RPE cells to epoxomicin and BafA significantly increased the ROS levels when compared with IL-1α-primed control cells (Figure 8A). The response was 1.1-fold significantly higher in MG-132-and-BafA-treated cells than in those exposed to epoxomicin and BafA (Figure 8A). MitoTempo significantly reduced the ROS levels in epoxomicin-and-BafA-treated cells (Figure 8B). Likewise, MitoTempo also reduced ROS levels upon MG-132-and-BafA treatment (Figure 8B). Together, our data suggest that both epoxomicin and MG-132 increase oxidative stress in human RPE cells and are part of the ROS derived from mitochondria.

## 4. Discussion

Dysfunctional proteasomes exert oxidative-stress-associated inflammation and contribute to multiple age-related degenerative diseases, including AMD [43,44,45,46,47]. Studies on proteasome-related oxidative stress and inflammation in disease progression widely used MG-132 as an inhibitor, but it is challenged by functionality on targets other than proteasomes as well [48,49,50,51,52,53]. Although several studies have shown differences in the specificity of proteasome inhibition mechanisms between MG-132 and epoxomicin [28,54,55], none of the studies demonstrated differences in inflammasome activation regarding MG-132 and epoxomicin. We have previously shown that MG-132 reduces cellular viability and activates NLRP3 inflammasome signaling with IL-1β secretion in human RPE cells [38]. According to our present data, the inhibition of proteasomes using epoxomicin resulted in higher levels of IL-1β than that using MG-132 treatment with comparable cytotoxicity. Similar to MG-132, the activation of caspase-1 appeared indispensable to IL-1β secretion, which points to inflammasome activation.

Several studies have shown NLRP3 inflammasome activation and inflammation in RPE cells and AMD progression, and NLRP3 has been found to be abundant in the RPE cells of AMD patients as well [56,57]. In addition to MG-132, contents of lipid aggregates, such as N-retinylidene-N-retinylethanolamine (A2E), carboxyethylpyrrole protein, malondialdehyde (MDA), 4-hydroxynonenal (4-HNE), and amyloid-β are also among the factors that have been shown to evoke NLRP3 inflammasome and cytokine production [58,59,60,61]. Our present data show that selective proteasome inhibition using epoxomicin resulted in AIM2 inflammasome-mediated IL-1β secretion in ARPE-19 cells, whereas the role of NLRP3 was insignificant. The previous finding of NLRP3 inflammasome activation by MG-132 [38] was verified here, but in addition, present results suggest that AIM2 inflammasome also participated in MG-132-induced IL-1β production. According to these data, MG-132 resembles UVB irradiation that also promoted the activation of both NLRP3 and AIM2 inflammasomes in RPE cells, but upon UVB exposure, AIM2 inflammasome dominated over NLRP3 [62]. 

In our previous study, oxidative stress dominated as the activation mechanism of inflammasome signaling, and the blockade of K^+^ efflux tended to reduce IL-1β release, but the effect was not statistically significant [38]. The present data clearly suggest that K^+^ efflux was involved in the IL-1β secretion by ARPE-19 cells upon both epoxomicin and MG-132 exposure. In addition, antioxidants NAC and APDC reduced cellular cytotoxicity and IL-1β levels in both epoxomicin- and MG-132-treated cells. This indicated that oxidative stress contributes also to the AIM2 inflammasome activation in RPE cells. The effect of NAC was significantly stronger with MG-132 than with epoxomicin, which suggests that oxidative stress is a more prominent mechanism in MG-132 exposure, which is in line with our previous findings [38]. The effect of APDC alludes to NADPH oxidase activity as the source of ROS, but the reduction in ROS levels when using MitoTempo refers to the role of mitochondria as an additional source of ROS. Oxidative-stress-related mitochondrial damage and mitochondrial-derived ROS have been elaborated in RPE degeneration [63,64,65,66]. 

Finally, studies have shown that proteasome degradation directly regulates mitochondrial protein turnover and mitochondrial energy metabolism [67,68,69,70,71]. Concurrently with epoxomicin exposure and increased mitochondrial ROS, our data show that dysfunctional intracellular clearance led to mitochondrial damage and subsequent release of mtDNA in the cytosol. mtDNA is a strong activator of the AIM2 inflammasome [32], and the present results support its role in the IL-1β maturation with epoxomicin. We have recently shown that dysfunctional intracellular clearance increased AIM2 gene expression in AMD patient-derived induced pluripotent stem cell (iPSC)-RPE cells [72], and induced mitochondrial damage activated the AIM2 inflammasome in ARPE-19 and D407 cells [73]. AIM2 inflammasome activation has also been shown in diseases such as psoriasis [74,75,76], atherosclerotic lesions [77], or cardiovascular diseases [78] that have ocular manifestations or serve as risk factors for AMD. Altogether, our data complement the mechanisms behind inflammation upon dysfunctional intracellular clearance in human RPE cells and emphasize the role of AIM2 alongside the NLRP3 inflammasome signaling.

## 5. Conclusions

**Figure 9:**
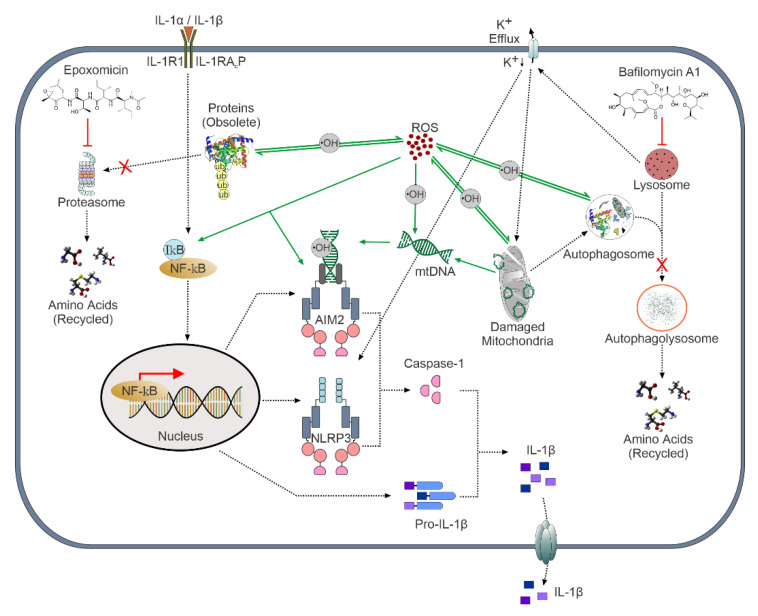
Epoxomicin-induced proteasome inhibition led to mitochondrial damage and ROS production, followed by activation of the predominant inflammasome AIM2 and, to a lesser degree, that of inflammasome NLRP3.

Altogether, our data present evidence of mtDNA-mediated AIM2 inflammasome activation and IL-1β secretion in human RPE cells upon exposure to the selective proteasome inhibitor epoxomicin. AIM2 may represent a novel therapeutic target in the prevention or treatment of AMD, and further studies on AIM2 inflammasome are highly justified. The schematic summary of the present study is presented in Figure 9.

## Figures and Tables

**Table 1:** Gene targets and primers used for mtDNA measurement.

Gene	Gene Accession Number	Sequence
mt-CYB	NC_012920.1	forward 5′-TCT CCG ATC CGT CCC TAA CA-3′
		reverse 5′-GTG ATT GGC TTA GTG GGC GA-3′
GAPDH	NM_001357943.2	forward 5′-ACA ACT TTG GTA TCG TGG AAG-3′
		reverse 5′-GCC ATC ACG CCA CAG TTT C-3′

## Data Availability

Data is contained within the article.
